# Multidimensionality of Health Inequalities: A Cross-Country Identification of Health Clusters through Multivariate Classification Techniques

**DOI:** 10.3390/ijerph15091900

**Published:** 2018-09-01

**Authors:** Javier Alvarez-Galvez

**Affiliations:** Department of Biomedicine, Biotechnology and Public Health, University of Cadiz, Avda. Ana de Viya, 52, 11009 Cádiz, Spain; javier.alvarezgalvez@uca.es; Tel.: +34-956-019-080

**Keywords:** health inequalities, social determinants of health, quantitative methods, cluster analysis, discriminant analysis

## Abstract

Despite major efforts in scientific literature to explain and understand the social determinants of health inequalities, the complex association between social causes and health outcomes remains empirically questionable and theoretically puzzling. To date, the studies on social determinants of health has mainly been generated by research techniques and methods that were developed to answer specific questions about the causes and effects of particular indicators on specific health outcomes. The present exploratory study follows a complex system approach to capture the interdependence between socioeconomic status, lifestyles, and health in a single measure that enables international comparisons of population health. Specifically, this study is aimed to: (a) classify individuals’ state of health according the usage of multidimensional data on physical and mental health, SES, lifestyles and risk behaviors, in order to (b) compare the relative strength of the different predictors of health groups (or clusters) at the individual-level and, finally, (c) to measure the level of health inequalities between different countries. From a complex system approach, this study uses multivariate classification methods to compare health groups in a sample of 29 countries and shows that interdependence models may be useful to describe and compare between-country health inequalities that are not visible through techniques for the analysis of dependence. The present work offers two fundamental contributions. On the one hand, this study compares the relative relevance of different indicators that are susceptible to affect individual health outcomes; on the other hand, the resulting multidimensional classification of countries according health clusters provides an alternative for inter-country health comparisons.

## 1. Introduction

Socioeconomic circumstances determine our lifestyles and health throughout life [1,2,3,4,5]. Structural conditions in which we live determine our individual behavior and our physical and mental health [6,7,8]. Studies shows the existence of widespread health inequities in post-industrialized societies that are closely related with differences in household income distribution, social class, occupational status, and educational attainment [9]. Findings has revealed that the higher the socioeconomic status (SES), the lower the prevalence of health problems, illness, disease and death [10,11]. Previous evidence has also indicated that poor socioeconomic conditions are commonly linked with intermediary determinants of health such as harmful lifestyles and risky behaviors [12]. Unhealthy diets and low physical inactivity have been also found negatively associated to physical and mental health [11,12]. For instance, studies show that regular physical activity reduces the risk of developing diabetes and metabolic syndrome—a cluster of conditions such as increased blood pressure, high blood sugar, abnormal cholesterol/triglyceride levels, and excess of fat around the waist [13]. Therefore, physical activity can prevent obesity, heart disease, and stroke, which are also related to a lower intake of vegetables and fruit [14], unhealthy habits such as smoking tobacco [15,16,17,18,19], and misusing alcohol or other noxious substances [20,21] that may increase the likelihood of having poor health.

In addition, studies have demonstrated that the predictive power of different social determinants of health varies between and within countries [2,22], but similarly variations have been found between different welfare state regimes characterized by distinctive social and health policies [3,23,24,25]. In epidemiological and public health literature, we can find multiple studies that confirm that the relationships between health, lifestyles, and socioeconomic status (SES) are contextually dependent. For instance, the effect of income household on poor health outcomes is lower in countries characterized by a universal public health system [2], and alcohol misuse is more problematic among Eastern countries [21]. Thus, the association and relevance of these diverse social determinants may vary in different contexts [26]. In other words, we can say that the relationship between structural and intermediary determinants of population health might significantly change depending on the context, which make it difficult to understand the link between the wide set of possible social determinants and its global impact at the macro-level.

Despite major efforts in scientific literature to explain and understand the determinants of health inequalities, the complex association between social causes and health outcomes remains empirically questionable and theoretically puzzling [27]. To date, studies on social determinants of health has mainly been generated by research techniques and methods that were developed to answer specific questions about the causes and effects of particular indicators on specific health outcomes [28]. Statistical models in epidemiological and public health studies have commonly used regression models to describe causal relationships between health predictors and outcomes [29]. The problem is that the available empirical evidence commonly assumes a certain kind of bivariate directionality between single predictors and specific health results that, theoretically, could be separately described, foreseen, and understood [26]. Therefore, from this analytical point of view, we cannot describe how a wide set of interdependent social determinants are connected in a single whole and how they affect poor health and health inequalities at the micro and macro level. The literature lacks studies that, on the one hand, describe the relative importance of the multiple dimensions that are susceptible to define population health (e.g., mental and physical state, diet, physical exercise, tobacco, alcohol, disability, SES, age, or gender, among many other), and on the other, how these diverse determinants might characterize health at the country level.

To fill this scientific gap, the present exploratory study follows a complex system approach to capture the interdependence between socioeconomic status, lifestyles, and health in a single measure that enables international comparisons of population health. Specifically, this study aims to: (a) classify individuals’ state of health according the usage of multidimensional data on physical and mental health, SES, lifestyles and risk behaviors, in order to (b) compare the relative strength of the different predictors of health groups (or clusters) at the individual level and, finally, (c) to measure the level of health inequalities between different countries.

These specific objectives are based on the following hypotheses:

**Hypothesis** **1.** *Individuals’ health can be systematically grouped in health clusters according quantitative similarities in multidimensional data (i.e., physical and mental health, lifestyles, risk behaviors, SES).*

**Hypothesis** **2.** *The predictive power of indicators associated with specific dimensions that compose health groups (i.e., mental and physical health, lifestyles, risk behaviors, SES) should present a relatively similar relevance in the final classification.*

**Hypothesis** **3.** *This multidimensional classification should provide an adjusted description of health groups that are more prevalent in different countries.*

## 2. Materials and Methods

### 2.1. Data and Variables

This study is based on the International Social Survey Programme (ISSP) 2011—“Health and Health Care” Study—(ZA 5800). This dataset has a sample size of 45,563 units at the individual level. The target population of this survey was individuals 18 years old and over. According routine data collection of each country, some exceptions were: Denmark (18–79), Finland (15–74), Japan (16 years old or older), Norway (19–78), Sweden (18–80), and South Africa (16 years of age or older). The ISSP Health covers information from 29 countries: Australia (AU), Belgium (BE), Bulgaria (BG), Chile (CL), Croatia (HR), the Czech Republic (CZ), Denmark (DK), Finland (FI), France (FR), Germany (DE), Great Britain (GB-GBN), Israel (IL), Japan (JP), The Republic of Korea (KR), Lithuania (LT), the Netherlands (NL), Norway (NO), Philippines (PH), Poland (PL), Portugal (PT), the Russian Federation (RU), Slovakia (SK), Slovenia (SI), South Africa (ZA), Sweden (SE), Switzerland (CH), Taiwan (TW), Turkey (TR) and the United States (US). Additional information on the ISSP Health and Health Care module can be found here: https://www.gesis.org/issp/modules/issp-modules-by-topic/health-and-health-care/.

Fourteen predictors of health outcomes have been used in this study: (a) variables related to “physical and mental health”, such as 1—health problems, 2—bodily aches or pains, 3—physical disability, 4—felt unhappy and depressed, 5—lost confidence, 6—not overcome problems (response categories are: 1 “Never”, 2 “Seldom”, 3 “Sometimes”, 4 “Often”); (b) “risky/healthy behaviors” such as 7—smoking habits (1 “Do not smoke and never did”, 2 “Do not smoke now but smoked in the past”, 3 “1–5 cigarettes per day”, 4 “6–10 cigarettes per day”, 5 “11–20 cigarettes per day”, 6 “21–40 cigarettes per day”, 7 “More than 40 cigarettes per day”), 8—alcohol consumption (1 “Never”, 2 “Once a month or less often”, 3 “Several times a month”, 4 “Several times a week”, and 5 “Daily”), 9—physical activity (1 “Never”, 2 “Once a month or less often”, 3 “Several times a month”, 4 “Several times a week”, and 5 “Daily”), 10—eat fresh fruit and vegetables (1 “Never”, 2 “Once a month or less often”, 3 “Several times a month”, 4 “Several times a week”, and 5 “Daily”), 11—Body Mass Index (BMI) (continuous variable derived from respondents’ height and weight: *IMC = kg/m*^2^); (c) “SES” defined as 12—socio-economic Index of Occupational Status (ISEI) (scale from 16 to 90, where 16 indicates the lowest SES and 90 the highest SES), and (d) “socio-demographic” determinants such as 13—respondent age (measured as a continuous variable) and 14—gender (0 “Male”, 1 “Female”). The ISEI index is computed using a causal model that, while controlling for respondents’ age, considers variables such as occupational status, education and income, and obtains scores for each occupation by the optimal scaling of the occupational unit group in the ISCO88 classification [30].

Commonly used socioeconomic variables, such as education or household income, were excluded from the analysis, since the ISEI index presented the advantage of being a continuous score, which integrates the effects of these indicators. Other variables such as marital status or ethnicity were omitted due to difficulties in comparing countries. Finally, self-rated health (SRH) was particularly used to test the accuracy of the resulting multidimensional classification; hence, this variable was also removed from the main analysis.

### 2.2. Statistical Methodology

Having tested the statistical significance of the indicators to explain health outcomes, a *k*-means cluster analysis was conducted to classify the state of health of the individuals in the sample into three basic categories: (1) bad health, (2) fair health, or (3) good health. Only three clusters were extracted in order to provide a main classification that could easily describe the resulting health profiles.

*K*-means clustering might be defined as a specific method of cluster analysis that aims to allocate *n* observations into *k* clusters, in which each observation belongs to the cluster with the nearest mean. Using this multivariate technique enables us to detect individuals with similar characteristics within a specific cluster, while at the same time these are distinguished from others with different attributes. Thus, the *k*-means clustering algorithm tends to find groups of comparable spatial extent. In *k*-means clustering, given a set of observations (*x*_1_, *x*_2_, …, *x_n_*), where each observation is a *d*-dimensional real vector, this method divides *n* observations into *k* clusters or groups (where *k ≤ n*) as disjoint subsets *S* = {*S*_1_, *S*_2_, …, *S_k_*} so as to minimize the within-cluster sum-of-squares.

The *k*-mean cluster method can be mathematically described as follows:

(1) $$\;\underset{s}{\arg\min\;}\overset{K}{\underset{i=1}{\sum }}\overset{n}{\underset{x_{j}\in S_{i}}{\sum }}{\left|\left|x_{n}-\mu_{i}\right|\right|}^{2}$$

where *x_n_* is a real vector representing the *n^th^* data point and *µ_j_* is the geometric centroid (i.e., the mean of data points) in *S_i_*.

Considering the sensibility of the *k*-means cluster technique when using different levels of measurement, all variables were previously standardized in the analysis to avoid problems in comparisons between the different scales of predictors. Once the variables were introduced in the analysis, we extracted these clusters in order to obtain a 3-group classification of general health. This division was aimed at ordering the initial dataset according a 3-part gradient in which respondents could be classified as having bad, fair or good health.

Finally, to evaluate the suitability of the classification and the explanatory power of the diverse indicators related with demographic characteristics, lifestyles, socioeconomic conditions and health outcomes included in the model, a study of validity was carried out using the discriminant analysis (DA) technique. DA is useful in determining whether a set of variables is certainly effective in predicting category membership (i.e., the final health group or cluster), and in particular to identify the indicators that might contribute more or less to the final health classification.

## 3. Results

Descriptive statistics for variables in the model are presented in Table 1. For a better interpretation of indicators in the model, variable labels for minimum and maximum values have also been provided.

### 3.1. Classification of Health Outcomes

Table 2 presents the final clusters through which individuals were classified as having: (1) bad, (2) fair or (3) good health. The results of the k-means cluster analysis allowed for observation of the composition of each of the obtained health group. The resulting scores in the final cluster centres revealed the average values of the variables included in the analysis for each of the clusters. Interpretation of the obtained scores was conducted using the standard deviation with respect to the mean, with the values above the average receiving a positive sign (+) and those below average, a negative sign (−). All indicators were found to be statistically significant in the cluster analysis. Table 3 provides a sematic interpretation of coefficients.

The cluster technique classified 25.8% of individuals with bad health, 30.7% with fair health, and 43.5% with good health. According to the health gradient of health clusters, individuals having a good state of health were characterized by low physical and mental health problems, low tobacco and alcohol consumption, significant physical activity, eating fresh food, low disability, low BMI, mid-young females with a mid-high socioeconomic status. On the other side, we found individuals reporting significant physical and mental health problems, low alcohol and tobacco consumption, low eating fresh food and physical activity, reporting certain disabilities, low weight, and old aged females with a mid-low socioeconomic position. In an intermediate position of health, mid-aged males with a medium socioeconomic status were characterized by a risky lifestyle presenting a higher consumption of tobacco and alcohol, low eating of fresh food, certain overweight compared with the other groups in the classification and reporting some disabilities. These results corroborate the internal validity of the resulting multidimensional health classification (H1).

### 3.2. Validation of the Classification

Sequential DA was conducted based on the resulting groups of the cluster analysis and the 14 individual variables that describe each of the groups. The results are shown in Table 4, where variables appear ordered in function of their overall discriminatory effect (composite potentiality index), and not for their individual contribution to each of the functions. For this reason, the composite potentiality index was calculated, resulting from the sum of the two simple indices of potentiality for each of the variables in the two discriminant functions.

The resulting discriminant model follows the requirements of the lower lambda values (near 0.0) and elevated *F* values. These criteria indicate the average differences between the variables of the compared groups, and a good cohesion between the members of the same group. The resulting discriminant functions and their structural coefficients show the suitability of variables in the model (only those values ≥0.30 are considered to be significant). Table 3 also includes the simple potentiality indexes, the statistical significance of the model and its relevance in terms of variance for the classification of the individuals in function of their health differences.

According to the second hypothesis, discriminant analysis shows the relevance of indicators related to mental health (“felt unhappy/depressed”, “not overcome problems” “lost confidence”), over the physical ones (“health problems”, “bodily aches and pains”). As is expected, gender is also a core factor in explaining the health classification of individuals in the sample. Indicators related to individual lifestyles (“BMI”, “alcohol” and “tobacco” consumption) are also relevant; however, these variables present a lower relevance in explaining the final clusters. Reporting disabilities also have statistically significant effects in the classification. However, variables such as “age”, “eat fresh”, “physical activity”, or “SES” are less relevant in the overall classification to explain individuals’ state of health and the final composition of health clusters (see structural coefficients <0.30 and the lower composite potentiality index) (H2).

**Figure 1:**
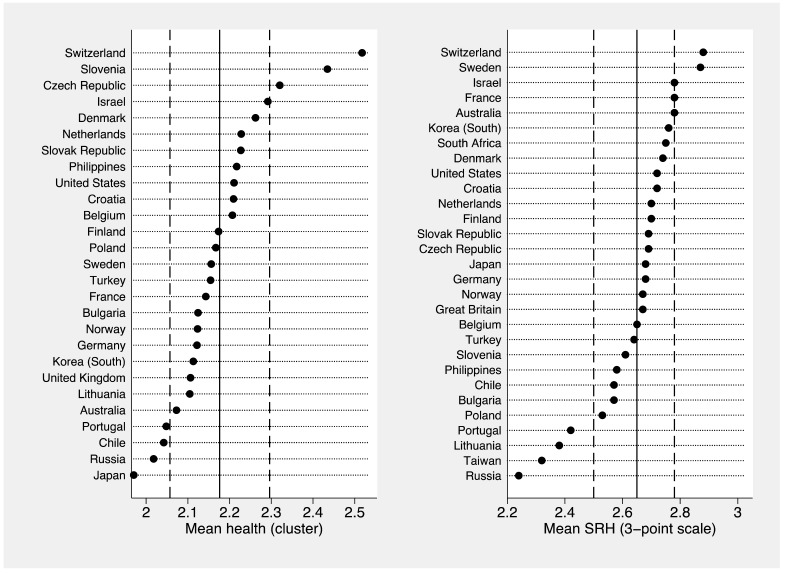
Inter-country comparison according the final three group classification (**left**) and self-rated health (**right**).

Once we confirmed the global validity of the classification and the internal relevance of every indicator in the model, the final health clusters were used to compare the relative position of the different countries, according the resulting health gradient. Taking into account that a 3-group health classification was extracted for countries in the sample, this grouping could be ordered on a 3-point scale (1 bad, 2 fair, and 3 good). These values provide information on the relative health position of every country, as an aggregated unit, according the initial classification of health groups based on a combination of multiple predictors of health (physical and mental health, risky behaviors and healthy habits, SES, and socio-demographic determinants). This inter-country comparison is presented in Figure 1 (left graph).

From the obtained 3-point scale defined by the clustering technique (1 “bad”, 2 “fair”, 3 “good” health), we observe that, in aggregated terms, the mean health of countries oscillates between the categories of “fair” and “good”. However, some countries seem to be better positioned around the average values (central lines) and standard deviations (dash lines). On the scale, Switzerland has the healthiest profiles, followed by Slovenia and Czech Republic, while Japan was found in the opposite position, followed by Russia, Chile, and Portugal. The remaining countries are distributed around the mean (2.157).

In order to validate the previously obtained classification, the variable SRH was used to compare the global position of countries according this simple measure (Figure 1, right graph). Considering that SRH is one of the most integrative predictors of general health and wellbeing, this indicator was used as a reference point. The initial SRH 5-point scale was transformed to a 3-point one for comparative purposes. The new scale was defined as 1 “poor” (formerly 1–2), 2 “fair” (formerly 3), 3 “good” (formerly 4–5). In general terms, we may observe that the isolated usage of SRH overestimates the general health of the population in every country, whereas our comprehensive index extracted through the cluster analysis presents a higher adjustment in relation to all the dimensions included in the analysis (H3). On the other hand, although there are concrete differences in the global health positioning of countries in the sample, both results present regularities that increase the external validity of this multidimensional approach to classify individuals around central health groups, and to describe and compare the prevalence of these health clusters at the country level (e.g., Switzerland or Israel at the top of the ranking; and Chile, Lithuania, Portugal or Russia at the bottom).

## 4. Discussion

The present exploratory study describes the relative relevance of multiple dimensions that are susceptible to explanation of health outcomes among different social groups, and uses a complex combination of variables to characterize health clusters at the individual and country level. The resulting model classifies people’s state of health according to the usage of a wide set of interdependent indicators related to structural and intermediary social determinants of health, identifying those that better define the resulting health clusters. Finally, this classification is used to measure and compare the countries that are better positioned, according this multidimensional health classification.

This study shows how people’s health is simultaneously shaped by multiple (structural and intermediary) social determinants of health that, combined in a particular way, determine both individual- and country-level health outcomes. As hypothesized, the predictive power of variables linked to specific latent dimensions that compose the final health clusters presents a similar importance in the classification. For instance, as we may observe in Table 4, the analogous composite index of mental and physical health indicators shows these factors to have a comparable predictive power to define the health clusters [1,5]; similar results can be found for other risk factors and socioeconomic determinants [2,26]. Indeed, the obtained classification supports this argument, since the resulting health groups offer a clear picture of the combination of indicators that might determine either a poor or a good health categorization. For instance, respondents reporting ‘good’ health were characterized by low physical and mental health problems, low tobacco and alcohol consumption, a significant physical activity, eating fresh food, low disability, low weight, mid-young females with a mid-high socioeconomic status, whereas people reporting mental or physical health problems were characterized by older females, low alcohol and tobacco consumption, low physical activity, presenting certain disabilities, low weight, and mid-low socioeconomic position.

As expected, the relevance of predictors explaining the resulting classification model is not the same. The variables that better characterize the model are those related to mental health (“felt unhappy/depressed”, “not overcome problems”, “lost confidence”), followed by physical health (“health problems”, “bodily aches and pains”) in the main discriminant function; however, in the second function, the indicators that present a higher relevance are respondents’ gender and those related to lifestyles. These findings indicate the presence of a higher gradient in mental health inequalities between countries in the sample and, in line with previous literature, the relevance of gender to define differences between individuals’ health. This might be possibly due to the higher variances in mental health between men and women [31]. On the other hand, physical health indicators are the following in terms of statistical relevance. This finding increases the validity of the classification since, according with previous studies, mental disorders have been found to be closely associated to the development of other physical health outcomes [10].

Variables related to lifestyles, and in particular those associated with unhealthy behaviors, such as alcohol and tobacco consumption, are also core predictors in the resulting health classification. However, compared to respondents’ gender and self-reported health variables, these indicators present a lower relevance in defining the final health clusters. Reporting disabilities also have significant effects on the classification, which reveals the importance of physical and mental health infirmities in defining the level profile (i.e., bad, fair, good) of individuals [32]. The last variables in terms of relevance for the classification are: “eat fresh”, “physical activity”, and “SES”. Clearly, later positioning in the composite potentiality index should not suggest that these indicators are no relevant to health outcomes; they only present a lower explanatory power when compared with other predictors that make up the final health clusters. In fact, at the individual level, their relevance in explaining health outcomes might vary depending on specific socio-cultural and educative characteristics of the population under study [33]. In any case, further research is needed to explain how socio-cultural characteristics of a population might impact the explanatory power of these indicators.

The final inter-country comparison provides information on the predominance of the three general health groups between different world regions. This study presents the advantage of identifying and comparing the resulting health classification in a wide and diverse set of countries. That is, according to the cluster technique, a characterization of health groups is obtained for all respondents in the sample, identifying which are the specific combination of attributes that describe their health profile. This classification is used to assign an individual and multidimensional score for every respondent in the sample, a value that finally is aggregated in an average score that measures the general level of health in each country according to the 3-point scale of health clusters. Compared to previous studies, this solution offers the advantage of obtaining a comprehensive measure of general health, which is used to compare inter-country health differences, instead of using univariate or bivariate descriptive measures that cannot integrate the interdependences between social determinants of health [28]. Therefore, the strengths of this study include, on the one hand, the identification and description of health groups and, on the other, the comparison of inter-country general health based on comprehensive and realistic health profiles that are based on multiple interrelated dimensions [34].

The results of this study give rise to several questions. On the one hand, countries such as Chile, Portugal, Russia or Japan are those that reveal higher differences in the resulting health score. These results are partially supported by previous studies that identify a higher prevalence of poor health outcomes in Eastern and Southern European countries [2,3,23,24,25]. However, the present study also identifies incongruences with previous literature; for instance, the poorer health outcomes in Japan (a country that, in fact, has low levels of health inequality compared to other East Asian countries such as South Korea) [35]. This result could be associated with the demographic structure in this country, but also cultural differences that might explain variations in self-reported health [36]. Clearly, this is a finding that should be studied at a deeper level. It highlights not only the necessity to increase the range of countries (and regions) that traditionally has been used in comparative health analysis (i.e., mainly the United States and the European countries), but also the fundamental requirement to increase the level of comparability among international survey data. On the other hand, at the methodological level, it is relevant to mention that additional socioeconomic measures (such as household income or education) should be used with the objective to test the effect of SES in the final classification and, in particular, to obtain alternative measures of identifying socioeconomic position. This might provide a better understanding of the impact of socioeconomic inequalities on health. In addition, although in the present study only three clusters were extracted in order to provide a classification that could easily describe the main health groups, further research could extend and contextually adapt this health gradient through the inclusion of additional health categories based on alternative indicators that are common in social and health survey data (e.g., life satisfaction, personal wellbeing, happiness, perceived discrimination, etc.).

Finally, the challenge for upcoming research will be to apply this approach using statistically representative data from different geographic areas at the meso-level, in order to analyze how these multiple dimensions might operate within specific countries. In addition, instead of self-reported health outcomes on physical and mental health, the incorporation of biomarker data in the future could also help us understand the link between social determinants and specific health outcomes (such as cardiovascular disease or cancer).

## 5. Conclusions

Although further work is required to disentangle the multiple interdependences between the social determinants of health, the present exploratory study represents a step forward in the description of the complex connection between structural and intermediary determinants of health. Using data from the ISSP Health Study and multivariate classification techniques, the present study shows that multidimensional models can be useful in synthesizing the effects of multiple interrelated indicators in a unique but more complex measure that makes it possible to compare and understand health results, both at the individual and country level. This study offers the advantage of combining cluster and discriminant analysis to identify and, subsequently, validate the health gradient in three main groups that integrate more complexity than the traditional cause-effect relationships. The findings highlight the importance of adopting a complex system approach in health policy and research to find a concrete combination of elements and mechanisms that might be essential for improving population health and reducing health inequalities.

## Figures and Tables

**Table 1:** Descriptive statistics.

Variable	Obs.	Mean	SD	Min	Max	Labels
Health problems	41,347	2.051	1.186	1	5	Never–Very often
Bodily aches or pains	42,691	2.487	1.222	1	5	Never–Very often
Felt unhappy-depressed	42,480	2.116	1.095	1	5	Never–Very often
Lost confidence	42,350	1.834	1.035	1	5	Never–Very often
Not overcome problems	42,337	1.872	1.054	1	5	Never–Very often
Smoke	43,848	2.026	1.423	1	7	Do not–40 per day
Alcohol	43,720	1.726	0.967	1	5	Never–Daily
Physical activity	44,311	2.955	1.389	1	5	Never–Daily
Eat fresh	44,959	4.256	0.929	1	5	Never–Daily
Disability	45,000	1.684	0.465	1	2	Yes–No
BMI	40,149	27.771	6.081	13.223	57.210	Metric variable
Gender	45,507	1.552	0.497	1	2	Male–Female
Age	45,385	48.235	17.434	16	102	Metric variable
SES (ISEI)	36,699	43.103	16.588	16	90	Metric variable

**Table 2:** Final cluster results.

Variable	1. Bad	2. Fair	3. Good
Health problems	1.01	−0.13	−0.51
Bodily aches or pains	0.92	0.02	−0.51
Felt unhappy-depressed	1.00	−0.22	−0.53
Lost confidence	0.98	−0.28	−0.46
Not overcome problems	0.99	−0.27	−0.51
Smoke	−0.02	0.53	−0.23
Alcohol	−0.23	0.72	−0.23
Physical activity	−0.17	0.18	0.12
Eat fresh	−0.04	−0.12	0.25
Disability	−0.63	−0.05	0.40
BMI	−0.10	0.72	−0.28
Gender	0.39	−0.98	0.36
Age	0.31	0.13	−0.15
SES (ISEI)	−0.21	0.03	0.20
Total	7003	8319	11,802
Percentage	25.8	30.7	43.5

**Table 3:** Description of cluster analysis results.

Variable	1. Bad	2. Fair	3. Good
Health problems	High	Mid-Low	Low
Bodily aches or pains	High	Mid	Low
Felt unhappy-depressed	High	Mid-Low	Low
Lost confidence	High	Mid-Low	Low
Not overcoming problems	High	Mid-Low	Low
Smoking	Mid-Low	High	Low
Alcohol	Low	High	Low
Physical activity	Mid-Low	Mid-High	Mid-High
Eating fresh food	Mid	Low	High
Disability	High	Mid	Low
BMI	Mid-Low	Higher	Low
Gender	Woman	Man	Woman
Age	Elderly	Mid age	Mid-Young
SES (ISEI)	Mid-Low	Mid	Mid-High
Total	7003	8319	11,802

**Table 4:** Discriminant analysis for validation of clustering technique and study of variables global relevance.

**Variable**	**Wilks’ Lambda**	**F ^I^**	**Discriminant Function 1**		**Discriminant Function 2**		
			**Structure Coefficient ^II^**	**Simple Potentiality index ^III^**	**Structure Coefficient**	**Simple Potentiality Index**	**Composite Potentiality Index ^IV^**
Felt unhappy depress.	0.587	9529.551	0.624 *	0.701	−0.036	0.002	0.702
Not overcome prob.	0.588	9485.402	0.621 *	0.694	−0.068	0.006	0.700
Lost confidence	0.613	8557.037	0.587 *	0.620	−0.091	0.010	0.630
Gender	0.614	8539.726	0.101	0.018	−0.713 *	0.612	0.630
Health problems	0.614	8528.289	0.591 *	0.629	0.009	0.000	0.629
Bodily aches pains	0.664	6868.175	0.525 *	0.496	0.096	0.011	0.507
BMI	0.805	3286.092	0.006	0.000	0.449 *	0.243	0.243
Alcohol	0.811	3161.640	−0.052	0.005	0.436 *	0.229	0.234
Disability	0.829	2791.683	−0.330 *	0.196	−0.092	0.010	0.206
Smoke	0.896	1573.633	0.029	0.002	0.309 *	0.115	0.116
Age	0.958	599.559	0.144 *	0.037	0.077	0.007	0.044
Eat fresh	0.967	461.337	−0.081	0.012	−0.136 *	0.022	0.034
SES (ISEI)	0.972	383.548	−0.123	0.027	−0.031 *	0.001	0.028
Physical activity	0.980	275.658	−0.097	0.017	0.054 *	0.004	0.020
**Global Relevance of the Discriminant Functions**							
**Canonic Correlation**		**Wilks’ Lambda**		**Chi-Square ^V^**		**% Correctly Classified**	
Function 1	Function 2	Function contrast 1 to 2	Function contrast 2	Function contrast 1 to 2	Function contrast 2	Original sample	Cross- validation
0.802	0.739	0.162	0.454	49336.812 (28)	21415.158 (13)	93.2	93.2

I. All of the *F* values were fully significant (0.000); II. Structural coefficients express the bivariate correlations of the independent variables (predictors) with the corresponding discriminant functions. Only those coefficients ≥0.30 are considered to be significant. From the squaring of these coefficients, we obtained the variance proportion of the variable that agrees with the discriminant function. The asterisk (*) indicates the absolute correlation of the variable with the function; III. The simple index of potentiality is obtained by multiplying the structural coefficient 2 by its relative eigenvalue in the discriminant function. The eigenvalue of function 1 is 1.800 (59.9%) and of function 2 is 1.203 (40.1%); IV. The composite potentiality index consists of the sum of the two simple potentiality indices for each of the functions. V. The model is perfectly significant (0.000), and the degrees of freedom are reported in parenthesis.
